# AI versus human-generated multiple-choice questions for medical education: a cohort study in a high-stakes examination

**DOI:** 10.1186/s12909-025-06796-6

**Published:** 2025-02-08

**Authors:** Alex KK Law, Jerome So, Chun Tat Lui, Yu Fai Choi, Koon Ho Cheung, Kevin Kei-ching Hung, Colin Alexander Graham

**Affiliations:** 1 The Accident and Emergency Medicine Academic Unit (AEMAU), The Chinese University of Hong Kong (CUHK), 2nd Floor, Main Clinical Block and Trauma Centre, Prince of Wales Hospital, Shatin, Hong Kong, China; 2 Department of Accident & Emergency, Tseung Kwan O Hospital, Hong Kong, China; 3 Hong Kong College of Emergency Medicine, Hong Kong, China

**Keywords:** Artificial intelligence, Educational measurement, Multiple choice questions, Medical education, Cognitive processes

## Abstract

**Background:**

The creation of high-quality multiple-choice questions (MCQs) is essential for medical education assessments but is resource-intensive and time-consuming when done by human experts. Large language models (LLMs) like ChatGPT-4o offer a promising alternative, but their efficacy remains unclear, particularly in high-stakes exams.

**Objective:**

This study aimed to evaluate the quality and psychometric properties of ChatGPT-4o-generated MCQs compared to human-created MCQs in a high-stakes medical licensing exam.

**Methods:**

A prospective cohort study was conducted among medical doctors preparing for the Primary Examination on Emergency Medicine (PEEM) organised by the Hong Kong College of Emergency Medicine in August 2024. Participants attempted two sets of 100 MCQs—one AI-generated and one human-generated. Expert reviewers assessed MCQs for factual correctness, relevance, difficulty, alignment with Bloom’s taxonomy (remember, understand, apply and analyse), and item writing flaws. Psychometric analyses were performed, including difficulty and discrimination indices and KR-20 reliability. Candidate performance and time efficiency were also evaluated.

**Results:**

Among 24 participants, AI-generated MCQs were easier (mean difficulty index = 0.78 ± 0.22 vs. 0.69 ± 0.23, *p* < 0.01) but showed similar discrimination indices to human MCQs (mean = 0.22 ± 0.23 vs. 0.26 ± 0.26). Agreement was moderate (ICC = 0.62, *p* = 0.01, 95% CI: 0.12–0.84). Expert reviews identified more factual inaccuracies (6% vs. 4%), irrelevance (6% vs. 0%), and inappropriate difficulty levels (14% vs. 1%) in AI MCQs. AI questions primarily tested lower-order cognitive skills, while human MCQs better assessed higher-order skills (χ² = 14.27, *p* = 0.003). AI significantly reduced time spent on question generation (24.5 vs. 96 person-hours).

**Conclusion:**

ChatGPT-4o demonstrates the potential for efficiently generating MCQs but lacks the depth needed for complex assessments. Human review remains essential to ensure quality. Combining AI efficiency with expert oversight could optimise question creation for high-stakes exams, offering a scalable model for medical education that balances time efficiency and content quality.

**Supplementary Information:**

The online version contains supplementary material available at 10.1186/s12909-025-06796-6.

## Introduction

High-quality multiple-choice questions (MCQs) are crucial for assessing medical trainees’ knowledge and ensuring safe clinical practice. The Hong Kong College of Emergency Medicine (HKCEM) conducts the Primary Examination on Emergency Medicine (PEEM) twice annually, requiring 100 newly constructed MCQs per exam for approximately 70 candidates. The rising reliance on high-stakes medical licensing exams has increased pressure on exam developers, possibly leading to burnout [1]. Traditionally, human experts create these MCQs, a time- and resource-intensive process [2, 3].

Preliminary studies have suggested that AI can efficiently generate a high volume of MCQs, potentially reducing the time and cost involved in traditional question creation [4, 5]. However, the ability of AI-generated MCQs to accurately assess medical knowledge, especially in the context of high-stakes professional examinations, remains underexplored. While existing literature often highlights the efficiency and practicality of AI in educational contexts, it lacks sufficient focus on the quality and psychometric robustness of AI-generated questions [6]. Most studies rely on anecdotal evidence or small-scale assessments of MCQs designed for low-stakes, formative, or undergraduate exams, often without the rigorous validation required to evaluate AI’s true potential [7].

For instance, Mistry et al. reported expert review scores of 9.8–9.9/10 for GPT-4-generated radiology MCQs, but the evaluation involved only two reviewers, raising concerns about subjectivity [8]. Similarly, small undergraduate studies (10–25 MCQs) reported acceptable discrimination indices (0.24–0.39), comparable to human-generated questions [9, 10]. However, these findings do not fully address AI’s suitability for high-stakes exams.

Moreover, research on ChatGPT-3.5 revealed a bias towards generating MCQs focused on lower-order cognitive skills, limiting their appropriateness for high-stakes assessments [11, 12]. No formal studies have yet examined ChatGPT-4o’s MCQs in relation to Bloom’s taxonomy, representing a key gap this study aims to address.

Furthermore, ChatGPT-4o represents a significant leap forward in AI capabilities, offering improved accuracy, context recognition, and the ability to handle more complex inputs. It is built on a larger and more diverse dataset than earlier versions, allowing it to generate more accurate and contextually appropriate responses [13]. Notably, ChatGPT-4o is 40% more likely to produce factually correct answers compared to its predecessors, and studies have demonstrated its superior performance in medical licensing exams, with accuracy rates reaching up to 90–100% on certain tests [14–16]. Also, compared to other large language models such as Claude 3 and Gemini 1.5, ChatGPT-4o has outperformed in medical knowledge assessments [17]. Despite these advancements, to existing knowledge, no study has rigorously tested ChatGPT-4o’s capabilities in direct comparison with human-generated questions in high-stakes examinations.

This study addresses these gaps by evaluating ChatGPT-4o’s ability to generate high-quality MCQs for professional medical licensing exams. It assesses psychometric quality, candidate performance, expert reviews, and time efficiency, offering a direct comparison with human-created MCQs in a professional setting.

## Methodology

This prospective cohort study involved medical doctors preparing for the Primary Examination on Emergency Medicine (PEEM), organised by the Hong Kong College of Emergency Medicine (HKCEM) in August 2024. The study population included doctors who had acquired basic medical degrees. They were recruited via email invitations sent to eligible candidates registered for the exam. The sampling technique was convenience sampling. Participation was voluntary, and candidates were required to provide informed consent. Individuals who did not complete both assessments or withdrew consent during the study were excluded.

The PEEM consists of 100 best-of-five multiple-choice questions designed to assess applied medical sciences topics such as anatomy, pathology, pharmacology, and physiology (25% in each category), all within the context of emergency medicine. This summative assessment serves as the gateway for entry into the basic emergency medicine specialty training programme in Hong Kong, making it a high-stakes examination.

Participants were given two sets of MCQs: AI-generated MCQs from ChatGPT-4o [18] and human-generated MCQs. The AI-generated MCQs were created using specifically tailored prompts for this study. ChatGPT-4o was instructed to generate a set of MCQs aligned with the key topics, learning objectives, and typical question formats of the PEEM examination. The same sample questions and MCQ writing guides provided to the human experts were also given to the AI system (the prompt and the generated MCQ are available with this link: https://chatgpt.com/share/67457f97-7aa4-8006-96b6-db36df06590f). Concurrently, a panel of 26 subject matter experts (human writing team) independently developed a set of 100 MCQs following the same guidelines as the PEEM examination, in line with the usual practice.

Both sets of multiple-choice questions underwent rigorous quality assessment by a panel of 6 expert reviewers who were not involved in the AI-generated and the human-generated-MCQ writing process (human review team). All of them were specialists in emergency medicine with more than 5 years of experience in medical education. The expert reviewers were not blinded to whether the MCQs were AI-generated or human-generated. This decision was intentional, as introducing blinding would have disrupted the standard PEEM production workflow. They evaluated the questions with a structured evaluation framework on five key aspects: factual correctness, relevance to emergency medicine, difficulty level, alignment with Bloom’s taxonomy cognitive levels [19] (examples can be accessed in electronic supplementary material), and the presence of item writing flaws [20]. Factual correctness was evaluated based on current medical guidelines and evidence-based practices. Relevance examines if the question addresses topics that are clinically appropriate for the PEEM context. Difficulty appropriateness evaluates whether the question’s challenge aligns with the expected knowledge level of the candidates sitting for the PEEM examination. Questions were considered duplicated if two or more MCQs asked about the same disease. The incidence of these problems in both the AI-generated and human-generated MCQ sets was systematically tallied to compare their prevalence. After independent reviews, the human review team convened to collectively evaluate the questions. Any differences in evaluation were discussed and resolved through consensus during the meeting.

Chat GPT-4o and the human writing team were asked to revise the MCQs. Feedback on the AI-generated MCQs was provided to ChatGPT-4o in an iterative process, replicating a dialogue-based approach. Comments from the expert review panel were copied directly into ChatGPT-4o to prompt revisions. For example, a comment such as, “Title: Q041… The stem mentions kidney, making all other distractors non-functional,” was provided as input to guide the AI in refining the question. Similarly, the human writing team received feedback and was allowed to make amendments until the questions met the required standards. Then, the human review team convened again to approve both sets of MCQ to ensure PEEM standards are met. Each team member logged the time spent upon task completion.

**Fig. 1:**
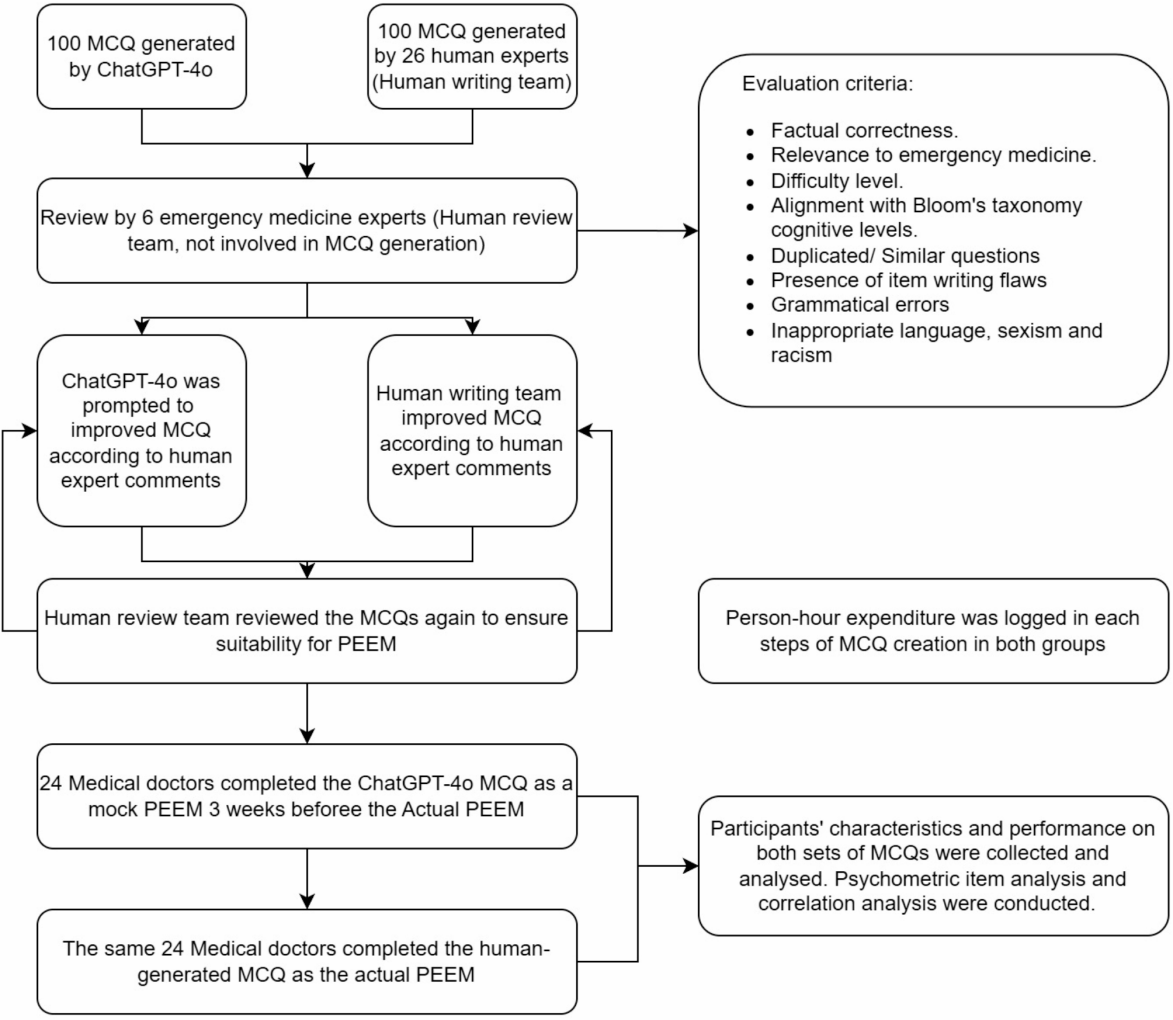
Study flowchart. MCQ: Multiple-Choice Question; PEEM: Primary Examination on Emergency Medicine

Participants were given the AI-generated MCQs through a mock examination conducted three weeks prior to the actual PEEM, which employed the human-generated MCQs. Both examinations followed the same format, structure, and assessment conditions to ensure consistency in candidate experience. Importantly, the same group of participants completed both the mock and the actual PEEM examinations, allowing for a direct comparison of performance across the two MCQ sets. Participants’ characteristics and performance on each MCQ from both sets were collected and analysed. Figure 1 summarises the conduction of the study.

### Sample size calculation

The sample size was calculated using G*Power software [21]. We employed an a priori t-test methodology, setting α at 0.05 and power at 0.8. In the absence of prior studies directly comparable to our research, we assumed an effect size (dz) of 0.5, representing a moderate effect. The calculated sample size was 34 participants.

### Statistical analysis

Descriptive statistics, including participant characteristics and candidate scores, were calculated for quantitative data. Psychometric item analysis involved evaluating the Difficulty Index, Discrimination Index, and Kuder Richardson Reliability (KR-20) statistic. These parameters were prioritised as they are widely accepted standards in psychometric analysis and are essential for ensuring both the validity and reliability of multiple-choice assessments. Difficulty Index, which equals the proportion of correct responses to each question, ranges from 0 to 1, with a higher value indicating easier questions. The Discrimination Index (DI) was assessed by comparing the percentage of correct responses between the top-performing 27% and the bottom-performing 27% of candidates. It measures how effectively a question differentiates between high- and low-performing candidates. For high-stakes medical examinations, such as professional licensing and specialty certification exams, a Discrimination Index (DI) ≥ 0.30 is considered good, indicating strong differentiation between high and low performers. A DI between 0.30 and 0.20 is generally acceptable, though not optimal, while a DI below 0.20 suggests marginal or poor discrimination. These thresholds served as the benchmark for evaluating both AI-generated and human-generated MCQs in this study [22]. Additionally, the KR-20 statistic was calculated to evaluate the internal consistency of the MCQs. Student’s t-tests were used to compare the indices between AI-generated MCQs and human-generated MCQs. A linear regression model was employed to explore the relationship between performance on the AI-generated MCQs and the human-generated MCQs. The correlation was calculated by Pearson’s correlation, and agreement between the two sets was assessed using the Intraclass Correlation Coefficient (ICC). A significance level of 0.05 and power of 0.8 were adopted using a two-tailed analysis. The statistical analyses were performed using R version 4.4.1 [23]. 

## Results

### Participant characteristics

Table 1 summarises the participant’s characteristics. The study involved 24 participants, yielding a participation rate of 64.9% from an invited pool of 37, of which 25 consented and one did not complete the AI-generated MCQs. Participants’ mean graduation years was 1.46 (SD= 2.00), with the distribution of their professional levels being 37.5% interns and 62.5% residents. The gender distribution was 33.3% female and 66.7% male. Age groups were primarily in the 25-29 range (54.2%).

**Table 1:** Participant characteristics

Years since graduating with a basic medical qualification	N	%
< 1	9	37.5
1	8	33.3
2	3	12.5
3	1	4.2
5	2	8.3
8	1	4.1
**Professional level**		
Internship	9	37.5
Resident	15	62.5
**Age**		
20–24	8	33.3
25–29	13	54.2

### Psychometrics item analysis

**Fig. 2:**
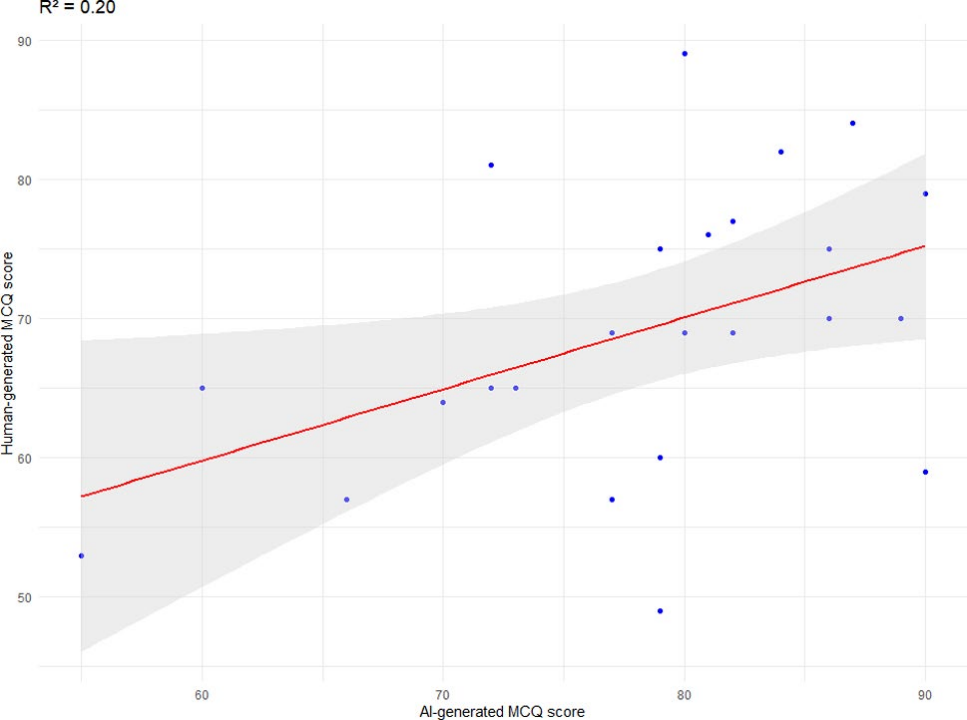
Scatter plot of AI MCQ and human MCQ performance score. Red line: best fitted linear regression model; shaded area: 95% CI

Table 2 summarises the psychometrics item analysis of the two sets of MCQs. The Difficulty Index for AI-generated MCQs was significantly higher (mean = 0.78, SD = 0.22) compared to human-generated MCQs (mean = 0.69, SD = 0.23), with a mean difference of 0.09 (95% CI = 0.03-0.15, *p* < 0.01), indicating that AI-generated MCQs were easier. However, there was no significant difference in the Discrimination Index between AI (mean = 0.22, SD = 0.23) and human MCQs (mean = 0.26, SD = 0.26). Both were considered acceptable but not optimal for high-stakes medical examinations. Notably, 51% of human-generated MCQs and 38% of AI-generated MCQs achieved optimal levels of discrimination (DI ≥0.3). The KR-20 values demonstrated good reliability for AI MCQs (0.75) and slightly better for human MCQs (0.83). The agreement between the two sets was moderate but statistically significant (Intraclass Correlation Coefficient [ICC] = 0.62, *p* = 0.01, 95% CI: 0.12–0.84). Pearson correlation (Fig. 2) between AI and human-generated MCQs performance was moderate but statistically significant (Pearson Correlation Coefficient, *r* = 0.45, Coefficient of Determination R² = 0.20, *p* = 0.03).

**Table 2:** Psychometrics item analysis

	AI MCQ	Human MCQ	Mean difference	95% CI	*p*
Difficulty index Mean +/- SD	0.78 +/- 0.22	0.69 +/- 0.23	0.09	0.03-0.15	<0.01
Discrimination Index Mean +/- SD	0.22 +/- 0.23	0.26 +/- 0.26	-0.04	-0.11-0.03	0.25
KR-20	0.75	0.83			

KR-20: Kuder-Richardson Formula 20 (a measure of internal consistency)

### Expert review

The expert review analysis of the MCQs, summarised in Table 3, revealed discrepancies. AI-generated MCQs had slightly higher instances of factual incorrectness (6% vs 4%), irrelevance to emergency medicine (6% vs 0%), and inappropriate difficulty levels for the PEEM (14% vs 1%), compared to human-generated MCQs. AI also generated more duplicated questions (14% vs 7%). Item writing flaws were slightly more prevalent in the AI-generated MCQs than the human-generated ones (37% vs 35%), with notable differences in ambiguous information and unnecessary content in the stems. Notably, the human-generated MCQs had more grammatical and spelling errors. The analysis of Bloom’s taxonomy cognitive levels revealed that AI-generated MCQs predominantly tested the ‘Remember’ and ‘Understand’ levels more frequently. In contrast, human-generated MCQs had a higher proportion of questions assessing the ‘Apply’ and ‘Analyse’ cognitive levels. (χ²=14.27, *p* = 0.003, examples of AI-generated MCQ in different Bloom’s taxonomy levels are shown in Appendix 1)

### Time expenditure analysis

Significantly less time was required for the overall creation of AI-generated MCQs (total 24.5 person-hours) compared to human-generated MCQs (total 96 person-hours). This difference was most pronounced in the initial writing phase, where AI required only 2 h for question generation, while humans spent 71 person-hours drafting questions.

**Table 3:** Expert review analysis

	AI MCQ	Human MCQ
**Factual Incorrectness**	6	4
**Irrelevance to Emergency Medicine**	6	0
**Inappropriateness for PEEM Level**	14	1
**Duplicated / similar question**	14	7
**Item Writing Flaws (IWF)**	37	35
Ambiguous or Unclear Information in the Stem	3	15
Negatively Worded Stems	0	0
Implausible Distracters	3	7
Unnecessary Information in the Stem	5	10
Multiple Correct Answers or No Correct Answer	11	1
Longest Option is Correct	0	0
Logical Cues in the Stem	6	0
Word Repeats in Stem and Correct Answer	6	0
Unfocused Stem	2	2
True/False Format Misused	0	0
Use of “All of the Above	0	0
Vague Terms like “Sometimes” or “Often”	0	0
Absolute Terms like “Never” or “Always”	0	0
Use of “None of the Above”	1	0
**Grammatical errors, spelling errors**	0	24
**Inappropriate language, sexism and racism**	0	3
**Bloom’s taxonomy cognitive level** (Chi-square= 14.27 *p*=0.003)		
Remember	47	28
Understand	37	28
Apply	13	26
Analysis	3	18
**Time expenditure (person-hour)**		
Planning, Blueprinting, and designing prompts for Chat GPT 4o	2.5	2
Writing	2	71
Review	15	17
Corrections	4	5
Editing	1	1
Total	24.5	96

## Discussion

The results of this study offer critical insights into both the potential and limitations of AI-generated MCQs within the context of high-stakes medical examinations. While AI-generated MCQs demonstrated significant time efficiency and non-inferiority in specific technical aspects, such as the discrimination index, they fell short in areas crucial for the educational effectiveness and integrity of medical assessments.

AI-generated MCQs were found to be significantly less challenging than those created by human experts. While this simplicity might be perceived as advantageous for assessing fundamental knowledge efficiently, it potentially limits the ability to evaluate more complex, nuanced understanding and application of medical concepts. The discrimination index (DI), which did not significantly differ between AI and human-generated MCQs, suggests both sets of questions were comparably effective in differentiating between high and low performers. In our study, the AI-generated MCQs achieved a mean DI of 0.22, although acceptable, is not ideal for high-stakes medical examinations. Only 38% of the AI-generated MCQ met the standard of DI ≥ 0.3. Similar findings were reported in studies using ChatGPT-3.5 [9, 10]. These findings suggest that while AI-generated MCQs show promise, further refinement is needed to enhance their discriminatory power, particularly in high-stakes assessment settings.

Although statistically significant, the ICC (0.62) and the Coefficient of Determination (R² = 0.20) observed in this study suggest limited agreement and predictive power between AI- and human-generated MCQs. While these values indicate a degree of consistency, they also highlight that AI-generated MCQs are not fully interchangeable with human-generated ones, particularly in high-stakes examination settings. The moderate ICC implies variability in how candidates responded to AI- versus human-generated questions, and the moderate R² suggests that performance on one set explains only a proportion of the variance in performance on the other. These findings underscore the importance of expert review and refinement to enhance the alignment of AI-generated MCQs with human-created standards.

The expert review of AI-generated MCQs raised significant concerns about their educational appropriateness. AI-generated questions showed a higher incidence of factual inaccuracies, with 6% of AI-generated MCQs containing errors. While this represents an improvement compared to the 30–40% error rate reported in studies using ChatGPT-3.5 [11, 24, 25],. it is important to note that these earlier studies often employed less optimised prompts, which may have contributed to the higher error rates rather than the inherent limitations of ChatGPT-3.5 itself [26]. Nonetheless, given the potentially negative educational consequences of incorrect information, human review remains essential. Additionally, our study identified a higher rate of irrelevant and inappropriately difficult questions in the AI-generated set, findings echoed by smaller studies [4, 11, 24]. These issues underscore the importance of rigorous editorial processes to ensure that AI-generated MCQs are both relevant and pedagogically sound.

Further analysis using Bloom’s taxonomy revealed a significant discrepancy in the cognitive levels assessed by AI and human-generated questions. AI-generated MCQs predominantly assessed lower cognitive skills (i.e., ‘Remember’ and ‘Understand’), whereas human-generated questions were more effective in evaluating higher-order cognitive skills such as ‘Apply’ and ‘Analyse.’ This aligns with previous studies [11, 24], which found that AI-generated questions tend to focus on lower cognitive levels. However, it is important to note that these studies, like ours, did not explicitly instruct the AI to prioritise higher-order cognitive skills in the prompts. Just as human experts require clear guidance to craft MCQs targeting higher-order thinking, AI also relies on well-designed prompts to achieve similar outcomes.

Despite these limitations, the significant reduction in time required to generate AI MCQs presents a clear advantage in terms of efficiency. AI-generated questions were created in a fraction of the time needed for human-generated MCQs, which could lead to substantial cost and time savings for educational institutions. Additionally, the absence of grammatical and spelling errors in AI-generated MCQs further streamlines the revision process, reducing the need for extensive proofreading and allowing for quicker deployment. This efficiency would allow educators to focus more on teaching and other high-value activities, although the trade-off in quality must be carefully managed.

The underlying differences in how ChatGPT-4o and human experts generate MCQs stem from the fundamental nature of artificial intelligence. ChatGPT-4o learns from patterns in vast datasets and generates content based on statistical likelihoods rather than an inherent understanding of the material. Mirzadeh et al. suggested that Large Language Models (LLMs) primarily operate through complex pattern matching rather than genuine logical reasoning [27]. This data-driven approach can lead to superficiality in question creation, especially in complex domains like medicine. Human experts, by contrast, draw on both explicit and tacit knowledge, which allows them to craft questions that challenge a range of cognitive skills and adapt to specific educational needs [28]. Furthermore, AI models, including ChatGPT, are susceptible to propagating errors or biases inherent in their training data [29, 30]. While AI can be fine-tuned, the technical process is less flexible and immediate compared to the adaptive and iterative nature of human question creation, where feedback can be quickly incorporated to improve the quality and relevance of the questions.

Improving the accuracy and cognitive level of AI-generated MCQs requires a combination of enhanced model training, expert collaboration, and innovative prompt engineering. Fine-tuning AI models with high-quality, domain-specific medical datasets, similar to how Medical Pathways Language Model (MedPaLM) incorporates medical knowledge from validated sources, could help produce more accurate and contextually relevant questions. Embedding instructions for case-based scenarios or critical thinking prompts which focuses on summarisation and synthesis, could elevate the cognitive complexity of questions. Incorporating complementary AI tools such as Perplexity AI for fact-checking and relevance assessment, along with a rigorous human review process, ensures quality and reduces inaccuracies. Regular updates with the latest medical guidelines and embedding real-world clinical scenarios into prompts can further align AI-generated MCQs with the requirements of high-stakes medical examinations.

Educators should also be aware that reliance on AI in high-stakes exams could raise concerns about fairness, accountability, and over-reliance on technology. AI outputs may reflect biases from training data, risking unintended disadvantages for certain candidate groups [31]. Clear accountability frameworks are needed to address inaccuracies and ensure oversight. Additionally, excessive dependence on AI might reduce educators’ engagement in question design. Transparent workflows, regular audits, and a balanced AI-human collaboration are essential to uphold fairness and educational integrity.

### Implications for future research and practice

We echo Inthrani et al. that integrating LLM tools like ChatGPT augments but does not replace human expertise [32]. With continual refinement, AI can produce high-quality questions, but the ultimate responsibility for quality and accuracy rests with subject matter experts, underscoring the irreplaceable role of human involvement in AI-driven education. Future research should focus on iterative AI-human collaboration models, where feedback loops are used to refine AI-generated MCQs progressively based on expert insights.

Furthermore, research should explore the integration of multimodal capabilities, such as incorporating diagnostic images or video into AI-generated questions, to test higher-order cognitive skills. Additionally, longitudinal studies examining the educational impact of AI-generated content on candidate performance in both low- and high-stakes settings would provide deeper insights into its efficacy and limitations. Finally, research into hybrid AI-human workflows could offer a scalable model for leveraging AI’s efficiency while maintaining content quality and relevance. Recent studies suggest that using AI to generate question templates, rather than fully formed questions, may be a more efficient and valid approach for integrating AI into the MCQ creation process [33].

Beyond MCQs, large language models hold potential for generating other assessment formats. For example, Script Concordance Tests, which evaluate clinical reasoning under uncertainty, have been explored in recent studies using LLMs. This suggests that AI can contribute to a broader spectrum of medical education assessments, potentially enhancing both the efficiency and quality of diverse question types [34].

### Limitations

One significant limitation of our study is the small sample size. Although a sample size calculation was performed with a target of 34 participants, only 24 were recruited. This discrepancy may result in an underpowered study. It is also important to note that the study participants were predominantly junior doctors, including interns and early-career residents. Their familiarity with foundational medical knowledge and relative inexperience with complex clinical reasoning tasks could have affected their performance on both AI- and human-generated MCQs. These factors could affect the generalisability of our findings. Moreover, the prompts for generating AI MCQs may have influenced the outcomes. While our prompts were carefully designed to align with PEEM standards, they did not explicitly emphasise higher-order cognitive skills. Also, the AI-generated questions were administered in a mock examination setting, while the human-generated questions were used for the actual PEEM examination. This difference in context may have influenced participant motivation and performance levels. Furthermore, there was a three-week gap between the two assessments, during which participants might have intensified their study efforts, potentially improving their performance on the actual PEEM examination. These factors represent potential confounding variables and should be considered when interpreting the results. Additionally, the AI model used in the study was only trained up to a specific cutoff date, and subsequent improvements in AI capabilities may not be reflected in the results. Finally, the lack of blinding of the expert evaluators and participants could introduce potential biases into the assessment process.

## Conclusion

ChatGPT-4o demonstrates significant potential for efficiently generating MCQs but requires expert oversight to ensure quality and alignment with educational standards. A hybrid AI-human framework is recommended, where AI handles the initial question generation and educators focus on review, refinement, and validation. Regular feedback loops and optimised prompt engineering should guide AI systems to produce more contextually appropriate and higher-order cognitive questions. Stakeholders in medical education should invest in structured workflows that balance AI efficiency with human expertise, ensuring that AI tools enhance rather than compromise assessment quality. Future research should focus on refining these collaborative models to ensure sustainable and scalable integration of AI into high-stakes examination processes.

## Electronic supplementary material

Below is the link to the electronic supplementary material.

Supplementary Material 1

## Data Availability

The datasets generated and/or analysed during the current study are available in the Mendeley repository, accessible at https://data.mendeley.com/datasets/n44nxxfymw/1.

## Abbreviations

AI: Artificial Intelligence
MCQ: Multiple-Choice Question
PEEM: Primary Examination on Emergency Medicine
HKCEM: Hong Kong College of Emergency Medicine
HKSEMS: Hong Kong Society for Emergency Medicine and Surgery
LLM: Large Language Model
DI: Discrimination Index
KR-20: Kuder-Richardson Formula 20 (a measure of internal consistency)
ICC: Intraclass Correlation Coefficient
CI: Confidence Interval
R²: Coefficient of Determination (used in regression analysis)
SD: Standard Deviation
