# Supplementary material for: AI versus human-generated multiple-choice questions for medical education: a cohort study in a high-stakes examination
Source: BMC Med Educ. 2025 Feb 8;25:208. doi: 10.1186/s12909-025-06796-6 (PMC11806894; doi:10.1186/s12909-025-06796-6)
Supplement: Supplementary file 1 — Supplementary Material 1 [file 12909_2025_6796_MOESM1_ESM.pdf]

Bloom’s Taxonomy Cognitive Level Examples

Remember:

Question:

A 60-year-old man presents with chest pain and shortness of breath. Which enzyme is most specific for diagnosing myocardial infarction?

Options:
a) Aspartate aminotransferase (AST)
b) Creatine kinase-MB (CK-MB)
***c) Troponin I**
d) Lactate dehydrogenase (LDH)
e) Myoglobin
Rationale: This question assesses the recall of specific factual knowledge about a diagnostic enzyme.

Understand

Question:
A 50-year-old patient is diagnosed with a peptic ulcer associated with *Helicobacter pylori* infection. How does *H. pylori* contribute to the development of peptic ulcers?
Options:
a) Direct invasion and destruction of the gastric mucosa
***b) Induction of chronic inflammation and impairment of mucosal defence mechanisms**
c) Production of toxins that directly cause epithelial cell necrosis
d) Inhibition of gastric acid secretion through histamine receptor antagonism
e) Formation of fibrous tissue in the gastric mucosa

Rationale: This question requires an understanding of the mechanism by which *H. pylori* contributes to peptic ulcer formation.

Apply

Question:
A 28-year-old man presents with sudden onset shortness of breath and chest pain. A CT scan confirms the presence of a pulmonary embolism (PE). Given the acute nature of the condition, which physiological change is most likely to occur as a DIRECT result of the pulmonary embolism?

Options:
a) Decreased cardiac output
***b) Increased pulmonary artery pressure**
c) Decreased alveolar dead space
d) Increased systemic vascular resistance
e) Decreased ventilation-perfusion ratio

Rationale: This question applies physiological knowledge to a clinical scenario involving pulmonary embolism.

Analyse

Question:
A 30-year-old woman presents with severe abdominal pain, diarrhoea, and fever. Stool culture grows a curved, gram-negative rod. Which organism is most likely responsible for her symptoms in the developed world?

Options:
a) *Escherichia coli*
b) *Shigella dysenteriae*
c) *Campylobacter jejuni*
d) *Salmonella enterica*
e) *Vibrio cholerae*

Rationale: This question requires the analysis of clinical symptoms, microbiological findings, and geographical context to determine the causative organism. It goes beyond simple recall and asks the candidate to correlate multiple pieces of information to arrive at the correct answer.
